# Between map and maze: reframing trust in healthcare AI

**DOI:** 10.1007/s00146-026-02997-9

**Published:** 2026-03-26

**Authors:** Hannah S. Piehl, Ricky Janssen, Bart Penders, Rianne R. R. Fijten

**Affiliations:** 1 Department of Radiation Oncology (Maastro), Research Institute for Oncology and Reproduction (GROW), Maastricht University Medical Centre, Maastricht, Netherlands; 2 Department of Health, Ethics & Society, Care and Public Health Research Institute (CAPHRI), Maastricht University, Maastricht, Netherlands; 3 Department of Health, Ethics & Society, Mental Health and Neuroscience Research Institute (MHeNs), Maastricht University, Maastricht, Netherlands

**Keywords:** Trust, Distrust, Mistrust, Artificial intelligence (AI), Healthcare, Scoping review

## Abstract

Artificial intelligence (AI) is often presented as a transformative technology for healthcare, promising to augment clinical decision-making, streamline workflows, and enhance diagnostic precision. Yet its integration into healthcare practice is shaped by the complex and often ambiguous notion of *trust*. While trust in AI has become a recurring theme across disciplines, there has been little systematic analysis of different conceptualizations of the term. This paper addresses this gap through an interdisciplinary scoping review that examines how trust, trustworthiness, distrust, and mistrust are articulated in literature on AI in healthcare. Drawing on 82 publications (2015–2025) retrieved from six databases (Web of Science, Scopus, PubMed, PhilPapers, SocINDEX, and ACM Digital Library), it maps how trust is defined, measured, or problematized, and identifies the conceptual, epistemological, and disciplinary boundaries that shape these approaches. The analysis puts forward five different conceptualizations: trust as a set of designable *principles*, trust as an *attitude* or *belief*, trust as a *binary* between cognition/affect or human/technology, trust as a *structural mechanism* within uncertainty, and trust as a *relational process* embedded in socio-technical systems. These framings illuminate how trust is treated as an attribute to be engineered, a behavior to be calibrated, or a relation to be cultivated—often removed from clinical practice. Yet, across literature, *trust disruptions* which manifest as distrust, mistrust, overtrust, and undertrust are undertheorized and are typically framed as obstacles to overcome rather than phenomena warranting analysis in their own right. By introducing these diverging conceptualizations, the paper argues that trust in healthcare AI is less a stable condition than a dynamic negotiation that reveals power relations, uncertainties, and institutional dependencies of AI. Recognizing distrust and mistrust as analytically productive allows for a more reflexive understanding of how trust is enacted, distributed, and contested within healthcare. This review contributes to an emerging body of work that reframes trust not as a prerequisite for technological adoption but as a contested and dynamic concept.

**Supplementary Information:**

The online version contains supplementary material available at 10.1007/s00146-026-02997-9.

## Introduction

Artificial intelligence (AI) is often presented as a transformative force in the healthcare sector, aimed at being integrated into many domains and use-cases (Shaw et al. 2019; Goirand et al. 2021). Trust affects how AI, a technology most cannot understand nor explain, travels through clinical practice. AI systems are developed and implemented under the (implicit) assumption that trust must be established for these technologies to function effectively (Bareis 2024; Lekadir et al. 2025). Research agrees that without trust, AI systems risk failure, whether in implementation or usage (Steerling et al. 2023; Hassan et al. 2024).

The concept of trust sits within longstanding intellectual traditions across sociology and philosophy. Sociological accounts, for example Simmel (1900) and Luhmann (1989), treat trust as a core social mechanism enabling action by reducing complexity and allowing humans to proceed despite uncertain situations. Moral philosophy highlights how trust rests on assumptions about another’s competence and willingness not to harm what matters to us (Baier 1986). In a different manner, O’Neill (2002) argues the focus should be on making institutions and agents trustworthy rather than on encouraging humans to trust more.

The fields of human-computer interaction and AI ethics extend these debates into the technological domain. Recent research on trust in AI systems, including healthcare applications, shows that user trust depends on contextual, user-specific, and system-level factors and that socio-ethical conditions shape trust relationships between users and AI systems (Bach et al. 2024; Lahusen et al. 2024). Central to these discussions is the argument that AI should be understood as a socio-technical system in which stakeholder relationships, perceptions of ethical conduct, and institutional arrangements influence perceived trustworthiness, acceptance, and adoption (Jasanoff 2004; Procter et al. 2023).

A recent systematic review by Malja and Afrasiabi (2025) highlights the social dimensions of AI, emphasizing that “AI does not operate in isolation; it interacts with and reshapes social institutions, power dynamics, and cultural norms” (p.2). Their typology identifies trust and transparency as central, linking both to explainability as ways to foster trust. This suggests that even work attentive to AI’s entanglement with social, cultural, and ethical structures treats mistrust as a knowledge gap solvable through explanation (Wynne 1992).

The topic of trust in AI systems has received growing attention from multiple disciplines, but its research has not yet been cohesively examined with attention to its different conceptualizations—especially not in the healthcare sector (Tucci et al. 2022; Albahri et al. 2023). Healthcare technologies have long mediated clinical decision-making, professional authority, and patient relationships (Robinson 2016; Hardon and Moyer 2014). This review situates AI within the broader ecosystem of these technologies, where it participates in existing dynamics of delegation, responsibility, and uncertainty. Yet its opacity, scalability, and perceived autonomy put forward new discussions around accountability and trust (Ananny and Crawford 2018). Studying trust in healthcare AI therefore offers insight into how established modes of expertise, decision-making processes, and patient-healthcare professional relationships are constituted. Healthcare provides a particularly illustrative context for examining how trust in AI is constructed and negotiated. Clinical decision-making occurs in a high-stakes environment, where emotional intensity, patient-centered responsibilities, and the potential for significant harm intersect. Moreover, preexisting professional hierarchies and institutional norms shape how trust is expressed, evaluated, and distributed among stakeholders. These factors complicate standard trust frameworks, revealing tensions and contingencies that may not be apparent in other domains.

## Objectives

Rooted in an interdisciplinary scoping review, this paper dives into how trust in healthcare AI is constructed, contested, and critiqued within the research landscape. Rather than presupposing trust, we explore how different disciplines and methodological approaches define and engage with trust in AI in healthcare. The divergent conceptualizations and disruptions of trust carry implications for how trust is approached not only in research but also in clinical practice.

This review of literature is guided by two research questions. First, we ask: how—from which perspectives and with which approaches—is trust in AI in the healthcare sector understood? Second, we ask: Which perspectives are missing in the research landscape of trust in AI in healthcare?

Without bringing divergent conceptualizations of trust into dialogue, research on healthcare AI risks obscuring how trust is shaped in clinical practice and who determines when a system is considered trustworthy. Design choices, professional responsibilities, and institutional guidelines all influence these judgments. A lack of conceptual clarity also has practical consequences: systems deemed trustworthy according to regulatory standards or technical criteria such as explainability, reliability, or transparency may still be met with hesitation in practice if their recommendations conflict with clinical intuition or established routines. Stakeholders may instead evaluate trustworthiness through legal liability, compliance metrics, or situational usefulness. These differing perspectives illustrate that trust in healthcare AI is contested, context-dependent, and shaped by multiple epistemologies.

By mapping how trust is constructed across disciplinary boundaries, this review aims to identify different framings as well as conceptual blind spots, contributing to a more reflexive and critical engagement with trust in AI within healthcare contexts. Our objective is a descriptive one: we do not advocate for all conceptualizations, but only aim to map, describe, and make explicit the ways in which trust, trustworthiness, and their disruptions are discussed.

## Methods

We conducted a scoping review using an interdisciplinary approach across six databases in order to include diverse disciplinary backgrounds and perspectives. We aimed at exploring the conceptually heterogeneous, interdisciplinary, and methodologically diverse research landscape instead of synthesizing comparable empirical outcomes. To address this in a structured yet flexible manner, a scoping review was more appropriate than a systematic review, meta-analysis, or narrative review (Levac et al. 2010).

**Fig. 1:**
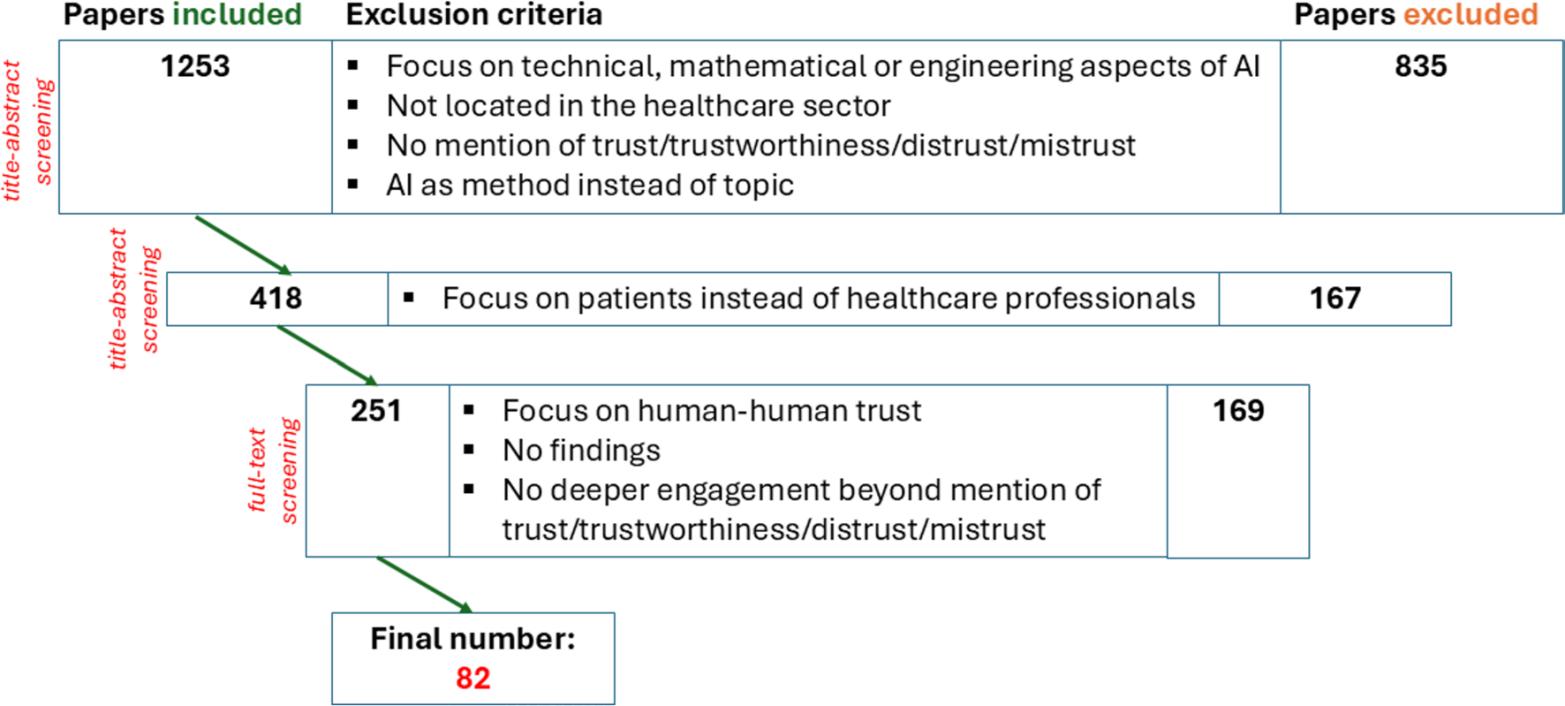
Screening process

We searched *Web of Science*, *Scopus*, *PubMed*, *PhilPapers*, *SocINDEX*, and *ACM Digital Library* using search strings iteratively developed with a university library information specialist (see appendix). The search focused on the three key concepts: trust, artificial intelligence, and healthcare. We prioritized papers with trust or trustworthiness in the title and also included those mentioning distrust or mistrust in the title, abstract, or keywords, as these often indicate deeper engagement with the concept. Included publications were available in full text, written in English, published between 2015 and January 2025, and categorized as scientific articles, position papers, preprints, or editorials. Screening was conducted manually using Rayyan.ai (https://www.rayyan.ai/) (Fig. 1).

After removing duplicates, 1253 publications were screened by title and abstract. Papers were excluded if they focused primarily on technical or engineering aspects of AI, were not situated in healthcare, did not engage with trust-related concepts (trust, trustworthiness, distrust, mistrust), or treated AI merely as a method rather than the object of inquiry. Because some databases did not allow searches restricted to titles, we additionally excluded papers that did not mention trust-related terms in the title unless distrust or mistrust appeared in the abstract or keywords. This screening resulted in 418 articles. To ensure consistent application of the criteria, RRRF, BP, and HSP independently screened the same random sample of 25 papers. Disagreements were discussed collectively and used to refine the exclusion criteria until consensus was reached. The remaining screening was then conducted by HSP. Consistent with the iterative nature of scoping reviews (Levac et al. 2010; Peters et al. 2015), we introduced an additional exclusion criterion. We narrowed the review to literature focusing on healthcare professionals, broadly defined as actors other than patients or members of the public. This reflects the central role of professionals in determining whether and how AI systems are implemented in clinical settings (Ayorinde et al. 2024; Boag et al. 2024). After this step, 251 papers remained for full-text screening.

During full-text screening, we excluded papers that examined trust solely between humans, lacked empirical or analytical findings, or mentioned trust only superficially without conceptual engagement. This process resulted in 82 included publications. The final set of publications was analyzed through inductive qualitative coding in ATLAS.ti (https://atlasti.com), focusing on conceptualizations of trust, definitions of trust and trustworthiness, references to legal or regulatory frameworks, and discussions of trust disruptions—understood as situations in which trust is challenged, compromised, or reconfigured. We then compared these codes to identify patterns as well as conceptual overlaps and tensions.

## Results and discussion

### Conceptualizations of trust and trustworthiness

Examining how trust is conceptualized in the reviewed literature reveals a range of perspectives, shaped by their disciplinary and methodological approaches. The five clusters of trust conceptualizations are not meant to be mutually exclusive but serve as lenses to surface tensions, overlaps, and combinations in how trust in healthcare AI is constructed. Fuzzy boundaries between those conceptualizations reflect the epistemic and practical messiness of trust, which our mapping highlights as a productive site for understanding how it is negotiated rather than categorically defined.

#### Trust through principles

The first cluster (30/82) conceptualizes trust as constituted through attributes of the AI system and its environment, with trustworthiness treated as an encompassing property. Attributes are typically formalized through principles (Hasani et al. 2022; El-Sappagh et al. 2023; Baldassarre et al. 2024). In this view, a trustworthy AI system is one that adheres to certain predefined qualities, for example reliability or dependability (Procter et al. 2023). These principles often appear in the form of lists, guidelines, or frameworks, suggesting that trustworthiness can be operationalized or measured by processes of quantification. Among these principles, explainability, transparency, understandability, validation, data quality and integrity are cited most frequently.

However, even if principles for a supposed trustworthy system are met, it does not follow that stakeholders will trust the system. Consequently, approaches need to also engage with how trust is understood and communicated in practice (Goisauf et al. 2025; Heuser et al. 2025). Moreover, conflicting ways to operationalize certain principles and meeting such criteria often introduce new complexities. For example, some publications equate explainability with trustworthiness, treating it as if providing explanations is sufficient to make a system trustworthy (Branley-Bell et al. 2020; Chanda et al. 2024). However, there are multiple ways and different levels of making a model explainable, which shows that principles are rarely universally defined (Markus et al. 2021). Therefore, meeting the principles of explainability to achieve trustworthiness is already more complex than often presented. This shows the limitations of principle-based approaches, which assume that certain attributes can be checked off to make a system trustworthy. Furthermore, a model may be explainable in a technical sense but not interpretable in clinical reasoning; validated statistically but not convincingly demonstrated in the lived realities of patient care; transparent at a system level but opaque at the moment of decision-making. Stakeholders may therefore judge a system as trustworthy in meeting predefined principles while withholding trust because it fails to meet relational, ethical, or moral expectations in practice.

#### Trust as a belief, trust as an attitude

The second cluster (12/82) understands trust as a belief or confidence in AI. This view shifts focus to the trustor’s *active* part of approaching the AI system. Often grounded in guidelines, performance criteria, or design principles, it connects the confidence to trust the system to the trustor believing in the system’s capabilities. This assumes that the trustor believes that a system is trustworthy or reliable if certain attributes are met. Similarly, many papers (19/82) also frame trust as an attitude of the trustor, emphasizing their *willingness* to trust (Fecho and Zöll 2023; van der Zander et al. 2024). In contrast to the former conceptualization of trust as principles, here, trust is put forward by the human towards the system. This means that the trustor has to believe that the qualities that are presumed to equate trust are legitimate or even actively decide to grant trust towards the AI system.

A subgroup of publications argues that trust can also be granted to untrustworthy systems, as trust ultimately depends on the human’s willingness to trust (van der Zander et al. 2024; Wünn et al. 2024). This reinforces the distinction between trust as a human action and trustworthiness as a property of the system. Other works highlight that the trustor must be willing to put themselves in a state of vulnerability (Branley-Bell et al. 2020; Pickering 2021; Fecho & Zöll 2023).

However, the processes in which this confidence is formed in practice are not explored further, as well as the role of different stakeholders in defining the principles on which trust is based. While this subgroup often uses technical language while referring to principles and guidelines, terms such as *good will* or *faith* are used as well, suggesting a tension between measurable attributes and more opaque, interpersonal dynamics (Winter and Carusi 2022; Zuchowski et al. 2024). Moreover, this approach to trust involves an active decision by engaging with the system, implying a level of responsibility for the trustor. It implicitly assumes they can either partially be blamed for their lack of willingness to trust or be held accountable for trusting the results of a system in cases where they should not have done so. Consequently, this places ethical responsibility on users—rather than on developers and the institutional structures that shaped the system’s design (Griffin et al. 2024). This superficial engagement, driven by technical or institutional stakeholders’ neglect of human relationships in clinical practice, could result in insufficient accountability of the structures and individuals shaping AI system development.

#### Binary conceptualizations of trust

The third cluster (7/82) puts forward binary conceptualizations of trust (Antweiler et al. 2024; Darvish et al. 2024). For example, cognitive trust is framed as *rational* and grounded in information about the system’s performance or functionality while affective trust is presented as *instinctive* and emotional. This dichotomy assumes that humans can separate *reason* from *affect* while making the decision to trust based on clearly differentiated assumptions.

A similar binary distinction comes up in discussions about trust versus reliability. In this framing, trust is something granted by the user, while reliability is something delivered by the system (Starke et al. 2022; Higgins et al. 2024). Accordingly, some scholars argue that AI systems, as non-human agents, cannot be trustworthy in a moral or ethical sense and that *reliance* is the more appropriate term (Procter et al. 2023).

These binary conceptualizations around trust insufficiently address the complexities that trust relations bring forward in healthcare. What counts as trustworthy is related to various factors, such as involved stakeholders, model characteristics as well as the social, institutional, and organizational context of AI. As these factors influence each other, a distinct separation between reason and affect is not possible in practice. Not taking the multiplicity of healthcare into account risks presenting political and infrastructural arrangements as neutral representations of practice (Bowker and Star 1999; Mol 2002). For example, sorting trust into cognitive and affective categories simplifies the complex and messy relations in which trusting processes take place and presupposes that trust can be explained, communicated, and then categorized from only one definite perspective.

#### Trust as a structure mechanism

The fourth cluster (11/82) conceptualizes trust informed by philosophical and sociological accounts. Here, trust functions as an implicit agreement, within which the trustor accepts vulnerability as part of a relationship with the trustee (El-Sappagh et al. 2023; Smith et al. 2023; De Proost and Pozzi 2024). The focus here lies on the relationship between the human and the system, where trust serves as a structure mechanism.

However, these conceptualizations often rely on abstract dyadic or triadic models which are intended to be applicable and transferable to most systems (Durán and Jongsma 2021; Ferrario and Loi 2022). This framing, often removed from practice, fails to acknowledge that trust is not only constituted between user and AI system, or in an extended manner, the AI developer. Consequently, this perspective overlooks broader socio-cultural and institutional contexts in which these relations are embedded.

In a similar manner, some papers view trust as a structure mechanism that materializes under conditions of uncertainty (Rey and Bouaynaya 2022; Starke et al. 2022; Higgins et al. 2024). Here, in the context of healthcare, riddled with complex decisions, trust functions as a necessary response to uncertainty, related to risk. This conceptualization implicitly takes the socio-technical system into account and includes expectations, norms, and evaluations circulating among peers, professional communities, institutions, and the broader public which shape what is considered trustworthy (Duenser and Douglas 2023). In practice, medical professionals already rely on implicit assumptions of trust in colleagues, institutions and technologies when making decisions (van Baalen and Carusi 2019).

#### Relational accounts of trust

The final cluster (15/82) situates trust within social and institutional contexts, recognizing its embeddedness in complex socio-technical systems—either implicitly or explicitly. These publications frame trust as co-produced through interactions and negotiations between diverse actors, highlighting the context-dependence of trust relations within healthcare AI. Maheshwari et al. (2024) give an account of institutional trust, describing how trust is embedded in a network “within and across institutions” (p.124) while Winter and Carusi (2022) focus on the context-dependent character of trust, highlighting that trust in AI cannot be separated from social practices.

These perspectives present more critical accounts of trust and move beyond techno-optimistic perspectives dominant in the current healthcare domain. Some papers take epistemological questions into account, while others focus on the practices around trust that shape the relationships of different actors and between human and AI (Neves et al. 2024; Winter and Carusi 2022). These perspectives highlight that trust in AI comes into being through material and social relations. Examining trust from a socio-technical system perspective provides a basis to questioning which perspectives are heard, understood and missing in the research landscape. This encompasses focusing on power relations and hierarchies of expertise within the field and understanding conceptualization of trust as ethically and politically charged.

### Trust disruptions

Investigating conceptualizations of trust also offers possibilities to engage with occasions where the linearity or conceptual clarity of trust is questioned, such as mentions of distrust and mistrust. We call these moments *disruptions of trust—*where trust is challenged, negotiated, or destabilized, either deliberately or inadvertently.

### Overtrust and undertrust

A group of publications puts forward the idea of *optimal* or *appropriate* trust (van der Zander et al. 2024). Model- and context-dependent, it carries the implication that there is a *right* amount of trust for each AI system and consequently frames deviations from appropriate trust as problematic. Additionally, many of these papers mention *overtrust* and *undertrust* (11/82). Overtrust refers to placing more trust in a system than justified, while undertrust means granting less trust than the system is judged to deserve (Pellikka et al. 2022; Kostick-Quenet al. 2024). These notions imply the existence of a universal standard for how much trust *should* be placed in AI systems and frequently appear in relation to discussions about over- and under-reliance as well as over- and under-use.

Closely related is the concept of *automation bias*, in which AI predictions are trusted over human judgment due to the assumption of objectivity in algorithmic outputs (Nickel 2022). This is also linked to *blind trust*, a term describing the phenomenon of AI outputs not being questioned (Högberg et al. 2024). However, AI is riddled with human assumptions and bias that cannot be separated from the system design (Friedman and Nissenbaum 1996; Selbst et al. 2019). Trust being granted to AI instead of human judgment is related to previously discussed binary notions of trust. Even when AI appears objective, uncritical reliance on AI simply shifts trust from humans to machines, maintaining binary distinctions, instead of capturing the complex, relational character of trust in practice.

*Misplaced trust* is conceptualized similarly normative in character. Seen as a core risk in medical AI deployment, it arises when initial trust is not reevaluated and reflected upon (Bürger et al. 2024). However, epistemological questions, such as who defines what *appropriate* trust looks like, remain unaddressed. Kostick-Quenet et al. (2024) distinguish between *actual* and *normative* trust, differentiating between how much stakeholders do trust versus how much they should trust the AI system. This resonates with the broader question of appropriate trust and again raises the issue of whose judgment determines what counts as appropriate, or normative.

*Trust calibration*, building on the logics of *over-* and *undertrust*, is defined as “appropriate trust judgment made by humans regarding the current state of AI capabilities and as a successful assessment of whether to follow or reject AI recommendations” (Naiseh et al. 2023, p.1). It is described as a corrective mechanism of *adjusting trust* and typically conceptualized as a cognitive process or grounded in value-based trust (Asan et al. 2020; Rojas et al. 2023; Darvish et al. 2024).

This narrative aligns with the idea of distinction between affective and cognitive trust. Regarding trust calibration, characterized by deliberation and critical assessment, it can be questioned whether trust can be *adjusted* or *calibrated* based on rational decision-making, or if it is intrinsically embedded in relational and affective dynamics which cannot be removed from this process. These framings imply that trust can and should be calibrated against an objective measure of the system’s capabilities, entailing that involved stakeholders can make informed decisions based on enough information as well as the existence of trustworthiness as a stable property of the AI system itself. However, *stable* trustworthiness does not exist in isolation of the social and human assessments through which it is enacted. Therefore, trust calibration does not only mean aligning trust with technical performance (criteria) but is carried out as a social process which the examined literature fails to acknowledge.

#### Distrust

A significant group of papers (17/82) mention *distrust.* Related to “lack of trust” (Diprose et al. 2020) or “extreme caution” (Kinney et al. 2024), distrust is often linked to human factors that cannot necessarily be influenced. For example, Kinney et al. (2024) refer to a “general distrust” towards AI, implying that distrust is not context-dependent nor processual but can be seen as a fixed property. Other works treat distrust as measurable, for instance by making use of a Likert scale, ranging from “distrust completely” to “trust completely” (Bussone et al. 2015).

There is also disagreement on what fosters trust or distrust. While Katzburg et al. (2024) argue that trust decreased through the provision of detailed information, Maheshwari et al. (2024) report distrust emerging through lack of information. This points to the fragility of trust calibration as a purely rational process. Even if a cognitive process of adjusting trust levels were possible, uncertainty regarding specific guidelines and principles supposedly fostering trust and distrust would still occur. Here, the conceptualization of distrust is removed from practice with a sole focus on theoretical considerations.

Some works distinguish between distrusting actions and attributes (Jones et al. 2023; Starke and Ienca 2024). In this case, *distrustworthiness* is treated as a property of the system, independent of whether users trust or distrust the system. This opens up the possibility of mapping mismatches where trust is either *deserved* or *undeserved*, *correctly placed* or *misplaced* (De Proost and Pozzi 2024), but also reinforces the notion of a fixed distrustworthiness in isolation of its human assessment, instead of coming into being through social relations.

Other publications describe a dependency relationship where individuals rely on AI systems despite distrust due to a lack of alternatives (Hallowell et al. 2022). In these discussions, trust-like behaviors are not rooted in trust but stem from distrust, emphasizing the entanglement of trust and distrust and the complexity of trust-relations. Furthermore, Hallowell et al. (2022) argue that distrust often arises from disagreement with the outcome or output of the AI system, which reinforces the idea of distrust as relational. Other works emphasize the processual character of distrust within environments of hope and negotiation, shaped by the experiences of the involved actors (Brown et al. 2024; Strange et al. 2024). Other publications highlight an institutional perspective. Here, the object of distrust is not only the AI system itself but the broader socio-political context in which it is embedded. Therefore, distrust becomes a form of critique directed at systemic structures rather than at technological artifacts (Kerasidou 2021). For example, De Proost and Pozzi (2024) argue that notions of systemic inequalities and structural injustices shape how distrustworthiness is constituted and repairing must encompass addressing epistemic justice issues within the medical sector.

Furthermore, one subgroup of papers introduces the notion of *healthy* or *legitimate* distrust, emphasizing that in certain contexts, distrust is both rational and welcomed (Strange et al 2024; Wolkenstein 2024). This puts forward discussions about agency, framing AI systems as a potential source of risk towards which distrust can be warranted. It also raises similar questions about the boundaries of legitimacy as the notion of calibrated trust. In this instance, identifying the boundaries of justified and unjustified, healthy or unhealthy distrust also becomes about determining specific criteria or variables.

Across literature, *distrust* appears as a frequently mentioned but rarely theorized concept. As the term functions more descriptively than analytically, we view this as a conceptual blind spot. While some publications focus on socio-technical or practice-focused dynamics around trust, distrust mostly remains detached from these conceptualizations in our body of literature. Consequently, distrust remains undertheorized and fragmented, overshadowed by the research landscape’s predominant focus on trust.

#### Mistrust and skepticism

It is unclear from the examined literature how conceptualizations of mistrust diverge from distrust or are simply used in its place. Brown et al. (2024) suggest the notion of refusal, relating it to the lived experiences of marginalized groups rejecting technologies as a political act rooted in histories of oppression. Zuchowski et al. (2024) highlight the betrayal of trust, which presupposes an initial state of trust that is violated in the process of engaging with technology. Relatedly, Pickering (2021) introduces the notion of *trust repair* as a response mechanism, “a willingness to identify issues, take responsibility to address them and contextualize behaviors within a narrative that makes sense to the trustor” (p.11).

Fecho and Zöll (2023) portray skepticism as an initial response to AI, which must be overcome by establishing trust. In contrast, Winter and Carusi (2022) and Chen et al. (2024) frame skepticism as a necessary response to experiences of blind trust or overtrust. This shows on a smaller scale how the examined approaches diverge on the question of whether skepticism, as well as distrust and mistrust, should be eradicated or can be seen as a constructive measure to approach an AI system. Finally, DeCamp and Tilburt (2019) argue that AI, given its non-human status, can only be relied on, not trusted, and thus should be met with distrust.

In conclusion, the literature reveals conceptual ambiguity surrounding mistrust towards healthcare AI, which carries connotations of agency, refusal, and resistance. Moreover, the distinction between mistrust and distrust remains undertheorized, reflecting a broader tendency in AI ethics and trust scholarship to overlook how mistrust could potentially serve as a productive concept to further investigate the relational dimensions around trust in healthcare AI.

### Limitations

This review has several limitations. First, literature on AI and trust in healthcare is exponentially growing. Studies published after our screening may present additional insights not captured here. Second, the scoping review was conducted primarily by one researcher (HSP), which may introduce selection and interpretive bias. Third, the thematic clusters presented are only one way to make sense of the field of trust in healthcare AI, alternative approaches could put forward different interpretations. Finally, this review did not include patient perspectives, which limits the understanding of trust from those affected by AI model outputs. Furthermore, excluding papers on human–human trust might have limited the number of relational or system-oriented conceptualizations of trust. Future studies could include multiple reviewers, incorporate patient perspectives, and explore alternative ways of organizing and interpreting the publication corpus to provide a more comprehensive account of trust in AI for healthcare. Furthermore, exploring how these different conceptualizations of trust manifest and intersect in practice (for example through a case study) represents a logical next step for research, moving beyond conceptual mapping toward a more practice-oriented understanding.

## Conclusion

We examined conceptualizations of trust to investigate the perspectives and approaches used to understand trust and trustworthiness. While we tried to introduce the nuances of the existing conceptualizations of trust in healthcare AI and its disruptions, the included papers can be divided in a specific way. The majority of publications (66/82) fail to take the socio-technical system in which trust is embedded into account, focusing instead on principles, technical differentiations, as well as human-centered notions of adjusting trust or measurements and valuations of trust. We do not argue that these approaches should be abandoned, but they are conceptually limited. They provide a map that offers guidance but cannot fully capture the uncertainties, negotiations, and relational dynamics encountered in practice. Our critique therefore aims to make the assumptions about trust within these approaches explicit, highlighting the limits of what they can capture. Principles, metrics, and checklists play an important role in evaluation and governance, but they do not operate independently of the relational, institutional, and normative contexts in which they are applied. Treating them as sufficient indicators of trustworthiness risks obscuring how trust is negotiated, contested, and unevenly distributed in practice.

The remaining papers (16/82) emphasize the relational and processual character of trust. For example, by conceptualizing trust as a series of negotiations and questioning its linearity and the checklist character of supposedly trustworthy systems. A relational perspective therefore reframes principles-based AI systems as neither inherently nor universally trustworthy, emphasizing that their meanings and effects depend on how they are interpreted, negotiated, and enacted within specific healthcare settings. Navigating trust in practice resembles traversing a complex maze rather than following a predetermined map, where progress emerges only through attending to multiple intertwined paths simultaneously, obstacles arise, and actors must continually reassess their direction.

Trust in healthcare AI is often framed as a tool for achieving implementation rather than examined as a contested normative idea. As Trench (2025) puts it, “trust is commonly presented as on–off, present or absent; in this representation, trust resembles faith or belief, rather than something that is considered and open to conversation” (p.329). Current research often overlooks the relational and contextual character of trust. Understanding trust in this way means recognizing that it cannot be engineered into a system but must be studied as part of the social and institutional relations that sustain or challenge it. Ethnographic observation, semi-structured interviews, and case studies on how trust comes into being in clinical practice can illuminate the relations around trust in a context-dependent manner, centering the mess and complexity that social science research puts forward (Law 2004; Sendak et al. 2020; Trench 2025). Such qualitative approaches do not provide a single measure of *trustworthiness* but instead show the relational and situational conditions under which trust is granted, withheld, or contested by different stakeholders, without reducing trust to a purely technical or individual attribute.

What counts as trustworthy varies considerably across professional and disciplinary boundaries. Different disciplinary perspectives and stakeholders invoke distinct epistemologies and diverging conceptualizations of trust. While they often remain isolated within individual publications, they intersect and collide in practice, rendering the field of healthcare AI messy, complex, and challenging to navigate. Consequently, these divergences have material consequences for how AI systems are designed, evaluated, and implemented. When one particular understanding of trust becomes dominant, others are marginalized. Not accounting for these conceptual blind spots potentially produces technologies that are *trustworthy* according to institutional metrics or technical principles but not aligned with the lived realities of healthcare professionals and patients.

This complexity is also visible in how literature discusses disruptions of trust. In fields such as implementation science, computer science, and health informatics, distrust is usually framed as a problem to be fixed or a barrier to overcome for the adoption of AI systems. In contrast, research in philosophy, sociology, and Science and Technology Studies views distrust as conceptually productive. These perspectives suggest that distrust can signal a moment for reorientation, revealing the assumptions, values, and inequalities that shape how AI systems are designed and used—or not used. Rather than seeing distrust as a failure of confidence, it can be understood as a signal of deeper tensions in how trust is distributed and justified. In these cases, established paths reach a dead end. Such dead ends force us to stop, retrace our steps, and reconsider technologically deterministic trajectories that frame AI implementation as a linear progression toward adoption. They make visible the limits of principles-based guidance and open spaces for reflection, renegotiation, and alternative pathways by understanding the conditions that made that path ineffective.

Compared to the research landscape’s attention towards trust, trust disruptions are undertheorized. This phenomenon extends beyond the field of healthcare AI towards other spheres of research, such as distrust in public science (Six and Latusek 2023). Moreover, Nowak et al. (2025) put forward the perspective that distrust in science is not the absence or opposite of trust but should be conceptualized on its own spectrum instead. Therefore, focusing on trust disruptions allows us to think about trust as an ongoing negotiation rather than a stable outcome. It highlights that trust and distrust are not opposites but interconnected processes that shape each other. This shift makes visible epistemologies of trust—whose trust matters, under what conditions, and with what consequences.

Making specific conceptualizations of trust, or the genealogy of the use of the term trustworthiness, explicit, could provide a sense of structure to the field. This could entail situating terminologies around trust in their specific disciplinary perspective and clinical context to put forward a more nuanced understanding and clearer research focus (Haraway 1988). Moreover, future research should investigate how trust and distrust are enacted in practice and how they come into being through interactions and negotiations. Investigating conflicts and tensions can serve as a constructive to not only deepen our understanding of trust as a socio-technical phenomenon but also support more responsible and context-aware forms of healthcare AI development and implementation in practice.

## Supplementary Information

Below is the link to the electronic supplementary material.

Supplementary file1 (DOCX 57 KB)

## Data Availability

No datasets were generated or analysed during the current study.
