# Supplementary material for: Between map and maze: reframing trust in healthcare AI
Source: AI Soc. 2026 Mar 26;41(7):6925–35. doi: 10.1007/s00146-026-02997-9 (PMC13385073; doi:10.1007/s00146-026-02997-9)
Supplement: Supplementary file 1 — Supplementary file1 (DOCX 57 KB) [file 146_2026_2997_MOESM1_ESM.docx]

Appendix

List of included publications

Abdelaal Y, Aupetit M, Baggag A, et al (2024) How Much Wearable Data is Enough for the Utility and Trust of Augmented Artificial Intelligence Systems? A Scenario-Based Interview with Medical Professionals. Int J Hum-Comput Interact. <https://doi.org/10.1080/10447318.2024.2400388>

Alelyani T (2024) Establishing trust in artificial intelligence-driven autonomous healthcare systems: an expert-guided framework. Front Digit Health 6:1474692. <https://doi.org/10.3389/fdgth.2024.1474692>

Antweiler D, Fuchs G, Fellner D., et al (2024) Extending the Visual Data Exploration Loop towards Trustworthy Machine Learning in the Healthcare Domain. Eurographics Association

Asan O, Bayrak AE, Choudhury A (2020) Artificial Intelligence and Human Trust in Healthcare: Focus on Clinicians. J Med Internet Res 22:e15154. <https://doi.org/10.2196/15154>

Baldassarre MT, Gigante D, Kalinowski M, et al (2024) Trustworthy AI in practice: an analysis of practitioners’ needs and challenges. Association for Computing Machinery, pp 293–302

Basharat I, Shahid S (2024) AI-enabled chatbots healthcare systems: an ethical perspective on trust and reliability. J Health Organ Manage. <https://doi.org/10.1108/JHOM-10-2023-0302>

Bélisle-Pipon JC, Powell M, English R, et al (2024) Stakeholder perspectives on ethical and trustworthy voice AI in health care. Digit Health 10:20552076241260407. <https://doi.org/10.1177/20552076241260407>

Bergquist M, Rolandsson B, Gryska E, et al (2024) Trust and stakeholder perspectives on the implementation of AI tools in clinical radiology. Eur Radiol 34:338–347. <https://doi.org/10.1007/s00330-023-09967-5>

Branley-Bell D, Whitworth R, Coventry L, Kurosu M. (2020) User trust and understanding of explainable ai: Exploring algorithm visualisations and user biases. Springer, pp 382–399

Brown P, van Voorst R, Brown P, van Voorst R (2024) The influence of artificial intelligence within health-related risk        work: a critical framework and lines of empirical inquiry. Health Risk Soc 26:301–316. <https://doi.org/10.1080/13698575.2024.2412374>

Browne JT, Bakker S, Yu B, et al (2022) Trust in Clinical AI: Expanding the Unit of Analysis. IOS Press BV, pp 96–113

Bürger VK, Amann J, Bui CKT, et al (2024) The unmet promise of trustworthy AI in healthcare: why we fail at clinical translation. Front Digit Health 6:. <https://doi.org/10.3389/fdgth.2024.1279629>

Burgess E, Jankovic I, Austin M, et al (2023) Healthcare AI Treatment Decision Support: Design Principles to Enhance Clinician Adoption and Trust. PROCEEDINGS OF THE 2023 CHI CONFERENCE ON HUMAN FACTORS IN COMPUTING SYSTEMS (CHI 2023). <https://doi.org/10.1145/3544548.3581251>

Bussone A, Stumpf S, O’Sullivan D, et al (2015) The role of explanations on trust and reliance in clinical decision support systems. Institute of Electrical and Electronics Engineers Inc., pp 160–169

Chanda T, Hauser K, Hobelsberger S, et al (2024) Dermatologist-like explainable AI enhances trust and confidence in diagnosing melanoma. Nat Commun 15:524. <https://doi.org/10.1038/s41467-023-43095-4>

Chen H, Ma X, Rives H, et al (2024) Trust in Machine Learning Driven Clinical Decision Support Tools Among Otolaryngologists. Laryngoscope 134:2799–2804. <https://doi.org/10.1002/lary.31260>

Choudhury A, Asan O, Medow JE (2022) Effect of risk, expectancy, and trust on clinicians’ intent to use an artificial intelligence system – Blood Utilization Calculator. Appl Ergon 101:. <https://doi.org/10.1016/j.apergo.2022.103708>

Darvish M, Holst J-H, Bick M, Bui T.X. (2024) Explainable AI in Healthcare: Factors Influencing Medical Practitioners’ Trust Calibration in Collaborative Tasks. IEEE Computer Society, pp 3326–3335

De Proost M, Pozzi G (2024) Why we should talk about institutional (dis)trustworthiness and medical machine learning. Med Health Care Philos. <https://doi.org/10.1007/s11019-024-10235-6>

DeCamp M, Tilburt JC (2019) Why we cannot trust artificial intelligence in medicine. Lancet Digit Health 1:e390. <https://doi.org/10.1016/S2589-7500(19)30197-9>

Diprose WK, Buist N, Hua N, et al (2020) Physician understanding, explainability, and trust in a hypothetical machine learning risk calculator. J Am Med Inform Assoc 27:592–600. <https://doi.org/10.1093/jamia/ocz229>

Durán JM, Jongsma KR (2021) Who is afraid of black box algorithms? On the epistemological and ethical basis of trust in medical AI. J Med Ethics. <https://doi.org/10.1136/medethics-2020-106820>

El-Sappagh S, Alonso-Moral J, Abuhmed T, et al (2023) Trustworthy artificial intelligence in Alzheimer’s disease: state of the art, opportunities, and challenges. Artif Intell Rev 56:11149–11296. <https://doi.org/10.1007/s10462-023-10415-5>

Ewals LJS, Heesterbeek LJJ, Yu B, et al (2024) The Impact of Expectation Management and Model Transparency on Radiologists’ Trust and Utilization of AI Recommendations for Lung Nodule Assessment on Computed Tomography: Simulated Use Study. JMIR AI 3:e52211. <https://doi.org/10.2196/52211>

Fecho M, Zöll A (2023) The Power of Trust: Designing Trustworthy Machine Learning Systems in Healthcare. Association for Information Systems

Ferlin, M., Klawikowska, Z., Niemierko J, et al (2023) Trustworthy Applications of ML Algorithms in Medicine - Discussion and Preliminary Results for a Problem of Small Vessels Disease Diagnosis. Springer Science and Business Media Deutschland GmbH, pp 3–16

Ferrario A, Loi M (2022) How Explainability Contributes to Trust in AI. Association for Computing Machinery, pp 1457–1466

Ferrario A, Loi M, Viganò E (2020) Trust does not need to be human: it is possible to trust medical AI. J Med Ethics 47:437–8. <https://doi.org/10.1136/medethics-2020-106922>

Gilbank P, Johnson-Cover K, Truong T (2020) Designing for Physician Trust: Toward a Machine Learning Decision Aid for Radiation Toxicity Risk. Ergon Des 28:27–35. <https://doi.org/10.1177/1064804619896172>

Gille F, Jobin A, Ienca M (2020) What we talk about when we talk about trust: Theory of trust for AI in healthcare. Intell Based Med 1:. <https://doi.org/10.1016/j.ibmed.2020.100001>

Goel K, Sindhgatta R, Kalra S, et al (2022) The effect of machine learning explanations on user trust for automated diagnosis of COVID-19. Comput Biol Med 146:105587. <https://doi.org/10.1016/j.compbiomed.2022.105587>

Goh WW, Chia KY, Cheung MF, et al (2024) Risk Perception, Acceptance, and Trust of Using AI in Gastroenterology Practice in the Asia-Pacific Region: Web-Based Survey Study. JMIR AI 3:e50525. <https://doi.org/10.2196/50525>

González-Gonzalo C, Thee EF, Klaver CCW, et al (2022) Trustworthy AI: Closing the gap between development and integration of AI systems in ophthalmic practice. Prog Retin Eye Res 90:101034. <https://doi.org/10.1016/j.preteyeres.2021.101034>

Grote T (2021) Trustworthy medical AI systems need to know when they don’t know. Journal of Medical Ethics 47:337–338. <https://doi.org/10.1136/medethics-2021-107463>

Hallowell N, Badger S, Sauerbrei A, et al (2022) “I don’t think people are ready to trust these algorithms at face value”: trust and the use of machine learning algorithms in the diagnosis of rare disease. BMC Med Ethics 23:112. <https://doi.org/10.1186/s12910-022-00842-4>

Hasani N, Morris MA, Rhamim A, et al (2022) Trustworthy Artificial Intelligence in Medical Imaging. PET Clin 17:1–12. <https://doi.org/10.1016/j.cpet.2021.09.007>

Higgins O, Chalup SK, Wilson RL (2024) Artificial Intelligence in nursing: trustworthy or reliable? J Res Nurs 29:143–153. <https://doi.org/10.1177/17449871231215696>

Högberg C, Larsson S, Lång K (2024) Engaging with artificial intelligence in mammography screening: Swedish breast radiologists’ views on trust, information and expertise. Digit Health 10:. <https://doi.org/10.1177/20552076241287958>

Jones C, Thornton J, Wyatt JC (2023) Artificial intelligence and clinical decision support: clinicians’ perspectives on trust, trustworthiness, and liability. Med Law Rev 31:501–520. <https://doi.org/10.1093/medlaw/fwad013>

Katzburg O, Roimi M, Frenkel A, et al (2024) The Impact of Information Relevancy and Interactivity on Intensivists’ Trust in a Machine Learning-Based Bacteremia Prediction System: Simulation Study. JMIR Hum Factors 11:e56924. <https://doi.org/10.2196/56924>

Kerasidou A (2021) Ethics of artificial intelligence in global health: Explainability, algorithmic bias and trust. J Oral Biol Craniofac Res 11:612–614. <https://doi.org/10.1016/j.jobcr.2021.09.004>

Kim M, Sohn H, Choi S, Kim S (2023) Requirements for Trustworthy Artificial Intelligence and its Application in Healthcare. Healthc Inform Res 29:315–322. <https://doi.org/10.4258/hir.2023.29.4.315>

Kim YJ, Choi JH, Fotso GMN (2024) Medical professionals’ adoption of AI-based medical devices: UTAUT model with trust mediation. J Open Innov: Technol Mark Complex 10:. <https://doi.org/10.1016/j.joitmc.2024.100220>

King H, Williams B, Treanor D, Randell R (2023) How, for whom, and in what contexts will artificial intelligence be adopted in pathology? A realist interview study. J Am Med Inform Assoc 30:529–538. <https://doi.org/10.1093/jamia/ocac254>

Kinney M, Anastasiadou M, Naranjo-Zolotov M, Santos V (2024) Expectation management in AI: A framework for understanding stakeholder trust and acceptance of artificial intelligence systems. Heliyon 10:e28562. <https://doi.org/10.1016/j.heliyon.2024.e28562>

Kitamura FC, Marques O (2021) Trustworthiness of Artificial Intelligence Models in Radiology and the Role of Explainability. J Am Coll Radiol 18:1160–1162. <https://doi.org/10.1016/j.jacr.2021.02.008>

Klincewicz M, Collins D, Jovanović IV, et al (2023) Institutional Trust in Medicine in the Age of Artificial Intelligence. In: The Moral Psychology of Trust. Lexington Books

Kostick-Quenet K, Lang BH, Smith J, et al (2024) Trust criteria for artificial intelligence in health: normative and epistemic considerations. J Med Ethics 50:544–551. <https://doi.org/10.1136/jme-2023-109338>

Larasati R, de Liddo A, Motta E (2023) Meaningful Explanation Effect on User’s Trust in an AI Medical System: Designing Explanations for Non-Expert Users. ACM Trans Interact Intelligent Syst 13:. <https://doi.org/10.1145/3631614>

Lee MH, Chew CJ (2023) Understanding the Effect of Counterfactual Explanations on Trust and Reliance on AI for Human-AI Collaborative Clinical Decision Making. Proc ACM Hum Comput Interact 7:. <https://doi.org/10.1145/3610218>

Lombardi A, Marzo S, Di Noia T, et al (2024) Exploring the Usability and Trustworthiness of AI-Driven User Interfaces for Neurological Diagnosis. ADJUNCT PROCEEDINGS OF THE 32ND ACM CONFERENCE ON USER MODELING, ADAPTATION AND PERSONALIZATION, UMAP 2024 627–634. <https://doi.org/10.1145/3631700.3665192>

Maheshwari K, Jedan C, Christiaans I, et al (2024) AI-Inclusivity in Healthcare: Motivating an Institutional Epistemic Trust Perspective. Camb Q Healthc Ethics 1–15. <https://doi.org/10.1017/S0963180124000215>

Naiseh M, Al-Thani D, Jiang N, et al (2023) How the different explanation classes impact trust calibration: The case        of clinical decision support systems. Int J Hum-Comput Stud 169:. <https://doi.org/10.1016/j.ijhcs.2022.102941>

Nasarian E, Alizadehsani R, Acharya U, et al (2024) Designing interpretable ML system to enhance trust in healthcare: A systematic review to proposed responsible clinician-AI-collaboration framework. Inf Fusion 108:. <https://doi.org/10.1016/j.inffus.2024.102412>

Neves BB, Omori M, Petersen A, et al (2024) Navigating artificial intelligence in care homes: Competing stakeholder views of trust and logics of care. Soc Sci Med 358:117187. <https://doi.org/10.1016/j.socscimed.2024.117187>

Nickel P (2022) Trust in medical artificial intelligence: a discretionary account. Ethics Inf Technol 24:. <https://doi.org/10.1007/s10676-022-09630-5>

Nundy S, Montgomery T, Wachter RM (2019) Promoting Trust Between Patients and Physicians in the Era of Artificial Intelligence. JAMA 322:497–498. <https://doi.org/10.1001/jama.2018.20563>

Pellikka PA, Hamza I, Carter RE (2022) What Is Needed for Artificial Intelligence to Be Trusted? Am J Med 135:421–423. <https://doi.org/10.1016/j.amjmed.2021.11.010>

Pelly M, Fatehi F, Liew D, Verdejo-Garcia A (2023) Artificial intelligence for secondary prevention of myocardial infarction: A qualitative study of patient and health professional perspectives. Int J Med Informatics 173:. <https://doi.org/10.1016/j.ijmedinf.2023.105041>

Pickering B (2021) Trust, but Verify: Informed Consent, AI Technologies, and Public Health Emergencies. Future Internet 13:. <https://doi.org/10.3390/fi13050132>

Prinster D, Mahmood A, Saria S, et al (2024) Care to Explain? AI Explanation Types Differentially Impact Chest Radiograph Diagnostic Performance and Physician Trust in AI. Radiology 313:. <https://doi.org/10.1148/radiol.233261>

Procter R, Tolmie P, Rouncefield M, et al (2023) Holding AI to Account: Challenges for the Delivery of Trustworthy AI in Healthcare. ACM Trans Comput-Hum Interact 30:. <https://doi.org/10.1145/3577009>

Quinn TP, Jacobs S, Senadeera M, et al (2022) The three ghosts of medical AI: Can the black-box present deliver? Artif Intell Med 124:102158. <https://doi.org/10.1016/j.artmed.2021.102158>

Rainey C, Bond R, McConnell J, et al (2024) Reporting radiographers’ interaction with Artificial Intelligence—How do different forms of AI feedback impact trust and decision switching? PLOS Digit Health 3:. <https://doi.org/10.1371/journal.pdig.0000560>

Rey A, Bouaynaya W (2022) Building Trust in Artificial Intelligence: Findings from Healthcare Organization. Association for Information Systems

Rojas JC, Teran M, Umscheid CA (2023) Clinician Trust in Artificial Intelligence: What is Known and How Trust Can Be Facilitated. Crit Care Clin 39:769–782. <https://doi.org/10.1016/j.ccc.2023.02.004>

Saboury B, Bradshaw T, Boellaard R, et al (2023) Artificial Intelligence in Nuclear Medicine: Opportunities, Challenges, and Responsibilities Toward a Trustworthy Ecosystem. J Nucl Med 64:188–196. <https://doi.org/10.2967/jnumed.121.263703>

Schwartz JM, George M, Rossetti SC, et al (2022) Factors Influencing Clinician Trust in Predictive Clinical Decision Support Systems for In-Hospital Deterioration: Qualitative Descriptive Study. JMIR Hum Factors 9:e33960. <https://doi.org/10.2196/33960>

Segers S, Mertes H (2022) The curious case of “trust” in the light of changing doctor–patient relationships. Bioethics 36:849–857

Shamszare H, Choudhury A (2023) Clinicians’ Perceptions of Artificial Intelligence: Focus on Workload, Risk, Trust, Clinical Decision Making, and Clinical Integration. Healthcare (Basel) 11:. <https://doi.org/10.3390/healthcare11162308>

Shevtsova D, Ahmed A, Boot IWA, et al (2024) Trust in and Acceptance of Artificial Intelligence Applications in Medicine: Mixed Methods Study. JMIR Hum Factors 11:e47031. <https://doi.org/10.2196/47031>

Silcox C, Dentzer S, Bates DW (2020) AI-Enabled Clinical Decision Support Software: A “Trust and Value Checklist” for Clinicians. NEJM Catal Inno Care Del 1:. <https://doi.org/10.1056/CAT.20.0212>

Sivaraman V, Bukowski L, Levin J, et al (2023) Ignore, Trust, or Negotiate: Understanding Clinician Acceptance of        AI-Based Treatment Recommendations in Health Care. PROCEEDINGS OF THE 2023 CHI CONFERENCE ON HUMAN FACTORS IN COMPUTING        SYSTEMS, CHI 2023. <https://doi.org/10.1145/3544548.3581075>

Smith MW, Kalsy M, Weir CR, et al (2023) Clinical Decision Support Systems and Trust in Automation: Case of a Clinical Reminder for Titration of Beta Blockers. SAGE Publications Inc., pp 1380–1385

Sperling J, Welsh W, Haseley E, et al (2025) Machine learning-based prediction models in medical decision-making in kidney disease: patient, caregiver, and clinician perspectives on trust and appropriate use. J Am Med Inform Assoc 32:51–62. <https://doi.org/10.1093/jamia/ocae255>

Starke G, Ienca M (2024) Misplaced Trust and Distrust: How Not to Engage with Medical Artificial Intelligence. Camb Q Healthc Ethics 33:360–369. <https://doi.org/10.1017/S0963180122000445>

Starke G, van den Brule R, Elger BS, Haselager P (2022) Intentional machines: A defence of trust in medical artificial intelligence. Bioethics 36:154–161. <https://doi.org/10.1111/bioe.12891>

Strange M, Ericson P., Khairova N., De Vos M. (2024) Beyond ‘Our product is trusted!’ – A processual approach to trust in AI healthcare. CEUR-WS, pp 59–68

Szabo L, Raisi-Estabragh Z, Salih A, et al (2022) Clinician’s guide to trustworthy and responsible artificial intelligence in cardiovascular imaging. Front Cardiovasc Med 9:1016032. <https://doi.org/10.3389/fcvm.2022.1016032>

van der Zander QEW, Roumans R, Kusters CHJ, et al (2024) Appropriate trust in artificial intelligence for the optical diagnosis of colorectal polyps: the role of human/artificial intelligence interaction. Gastrointest Endosc 100:1070-1078.e10. <https://doi.org/10.1016/j.gie.2024.06.029>

Wallace PJ (2024) Gaining Trust: Lessons and Opportunities for Artificial Intelligence in Health Care. Perm J 28:168–171. <https://doi.org/10.7812/TPP/24.064>

Winter P, Carusi A (2022) ‘If You’re Going to Trust the Machine, Then That Trust Has Got to be Based on Something’: Validation and the Co-Constitution of Trust in Developing Artificial Intelligence (AI) for the Early Diagnosis of Pulmonary Hypertension (PH). Sci Technol Stud 35:58–77. <https://doi.org/10.23987/sts.102198>

Wolkenstein A (2024) Healthy Mistrust: Medical Black Box Algorithms, Epistemic Authority, and Preemptionism. Camb Q Healthc Ethics 33:370–379. <https://doi.org/10.1017/S0963180123000646>

Wünn T, Sent D, Peute LWP, et al (2024) Trust in Artificial Intelligence: Exploring the Influence of Model Presentation and Model Interaction on Trust in a Medical Setting. Springer Science and Business Media Deutschland GmbH, pp 76–86

Yang Q, Hao Y, Quan K, et al (2023) Harnessing Biomedical Literature to Calibrate Clinicians’ Trust in AI Decision Support Systems. PROCEEDINGS OF THE 2023 CHI CONFERENCE ON HUMAN FACTORS IN COMPUTING SYSTEMS (CHI 2023). <https://doi.org/10.1145/3544548.3581393>

Zhan X, Abdi N, Seymour W, Such J (2024) Healthcare Voice AI Assistants: Factors Influencing Trust and Intention to Use. Proc ACM Hum Comput Interact 8:. <https://doi.org/10.1145/3637339>

Zhang J, Zhang ZM (2023) Ethics and governance of trustworthy medical artificial intelligence. BMC Med Inform Decis Mak 23:7. <https://doi.org/10.1186/s12911-023-02103-9>

Zuchowski LC, Zuchowski ML, Nagel E (2024) A trust based framework for the envelopment of medical AI. NPJ Digit Med 7:230. <https://doi.org/10.1038/s41746-024-01224-3>

Search strings

ACM Digital Library:

(Title:(trust* OR distrust* OR mistrust*) OR Abstract:(distrust* OR mistrust*)) AND (Title:("artificial intelligence" OR AI OR "machine learning" OR ML OR algorithm* OR "machine intelligence" OR "deep learning" OR "neural network*" OR computation* OR "decision support" OR "advisor* system*" OR "autonomous agent*" OR "intelligent system*")) AND (Title:(medic* OR health* OR clinic* OR physician* OR doctor* OR nurs* OR patient* OR care) OR Abstract:(medic* OR health* OR clinic* OR physician* OR doctor* OR nurs* OR patient* OR care))

PhilPapers:

PhilPapers only supports searches using the AND Boolean operator. As a result, we manually executed all 140 possible combinations of terms across the three conceptual categories.

PubMed:

(("Trust"[Mesh] OR trust*[Title] OR distrust*[Title/Abstract] OR mistrust*[Title/Abstract])) AND (("artificial intelligence"[Title] OR AI[Title] OR "machine learning"[Title] OR ML[Title] OR algorithm*[Title] OR "machine intelligence"[Title] OR "deep learning"[Title] OR "neural network*"[Title] OR computation*[Title] OR "decision support"[Title] OR "advisor* system*"[Title] OR "autonomous agent*"[Title] OR "intelligent system*"[Title]))

Scopus:

( TITLE ( trust* ) ) OR ( TITLE-ABS-KEY ( distrust* OR mistrust* ) ) AND ( TITLE ( "artificial intelligence" OR ai OR "machine learning" OR ml OR algorithm* OR "machine intelligence" OR "deep learning" OR "neural network*" OR computation* OR "decision support" OR "advisor* system*" OR "autonomous agent*" OR "intelligent system*" ) ) AND ( TITLE-ABS-KEY ( medic* OR health* OR clinic* OR physician* OR doctor* OR nurs* OR patient* OR care ) ) AND PUBYEAR > 2014 AND PUBYEAR < 2026 AND ( LIMIT-TO ( LANGUAGE , "English" ) )

SOCIndex:

(TI (trust* OR mistrust* OR distrust*) OR AB (trust* OR mistrust* OR distrust*) OR KW (trust* OR mistrust* OR distrust*)) AND (TI "artificial intelligence" OR ai OR "machine learning" OR ml OR algorithm* OR "machine intelligence" OR "deep learning" OR "neural network*" OR computation* OR "decision support" OR "advisor* system*" OR "autonomous agent*" OR "intelligent system*") AND (TI (medic* OR health* OR clinic* OR physician* OR doctor* OR nurs* OR patient* OR care) OR AB (medic* OR health* OR clinic* OR physician* OR doctor* OR nurs* OR patient* OR care) OR KW (medic* OR health* OR clinic* OR physician* OR doctor* OR nurs* OR patient* OR care))

Web of Science:

((TI=(trust*)) OR (TS=(distrust* OR mistrust*))) AND (TI=("artificial intelligence" OR AI OR "machine learning" OR ML OR algorithm* OR "machine intelligence" OR "deep learning" OR "neural network*" OR computation* OR "decision support" OR "advisor* system*" OR "autonomous agent*" OR "intelligent system*")) AND (TS=(medic* OR health* OR clinic* OR physician* OR doctor* OR nurs* OR patient* OR care))

Table of included publications

| **Reference** | **Publication type** | **Main concept** | **Study type** | **Discipline(s)** | **Application** | **Stakeholders** | **Application/ AI type** | **Trust Conceptualization** |
| --- | --- | --- | --- | --- | --- | --- | --- | --- |
| Abdelaal, Y., Aupetit, M., Baggag, A., Bashir, M., & Al-Thani, D. (2024). | journal article | trust | scenario-based interviews | human-computer-interaction (HCI) | diabetes | healthcare professionals | AI-generated recommendations for sleep and activity improvement and explanations of the underlying rationale | Structure Mechanism |
| Alelyani T. (2024). | journal article | trustworthiness | semi-structured interviews | Computer science, social computing |  | domain experts with extensive knowledge and experience in autonomous systems technologies in healthcare: development, evaluation, implementation | Autonomous Systems (AS) | Principles |
| Antweiler, D., Fuchs, G., Fellner D., Fellner D., El-Assady M., & Schulz H.J. (2024). | conference paper | trustworthiness | proposal of framework | applied computing |  | data scientists, visual analytics experts, healthcare professionals | visual data | Binary conceptualizations |
| Asan O, Bayrak AE, & Choudhury A. (2020). | position paper | trust | literature review | computer science |  | clinicians |  | Belief |
| Baldassarre, M. T., Gigante, D., Kalinowski, M., Ragone, A., & Tibidò, S. (2024). | conference paper | trustworthiness | survey, semi-structured interviews | software engineering; software creation and management |  |  |  | Principles |
| Basharat, I., & Shahid, S. (2024). | journal article | trust | semi-structured interviews | health sciences |  | patients, healthcare professionals, academic researchers, ethicists, and legal experts | AI-enabled chatbots | Relational |
| Bélisle-Pipon JC, Powell M, English R, Malo MF, Ravitsky V, & Bensoussan Y. (2024). | journal article | trustworthiness | questionnaire | health sciences |  | voice AI experts, clinicians, scholars, patients, trainees, policymakers | voice AI | Principles |
| Bergquist M, Rolandsson B, Gryska E, Laesser M, Hoefling N, Heckemann R, Schneiderman JF, & Björkman-Burtscher IM. (2024). | journal article | trust | semi-structured interviews | information technology, social science | radiology | healthcare professionals | AI applications in radiology | Principles |
| Branley-Bell, D., Whitworth, R., Coventry, L., & Kurosu M. (2020). | conference paper | trust | A-priori power analysis | HCI |  | healthcare professionals & other participants | diagnostic tool - supervised learning algorithms | Attitude |
| Brown, P., van Voorst, R., Brown, P., & van Voorst, R. (2024). | journal article/editorial | trust, distrust | Literature review/philosophical inquiry | critical social science/STS |  |  |  | Relational |
| Browne, J. T., Bakker, S., Yu, B., Lloyd, P., Ben Allouch, S., Schlobach S., Perez-Ortiz M., & Tielman M. (2022). | conference paper | trust | Literature review | HCI |  |  |  | Attitude |
| Bürger, V. K., Amann, J., Bui, C. K. T., Fehr, J., & Madai, V. I. (2024). | position paper | trust, trustworthiness | position | health sciences |  |  |  | Principles |
| Burgess, E., Jankovic, I., Austin, M., Cai, N., Kapuscinska, A., Currie, S., Overhage, J., Poole, E., Kaye, J., Burgess, E. R., Jankovic, I., Austin, M., Cai, N., Kapuscinska, A., Currie, S. T., Overhage, J. M., Poole, E. S., & Kaye, J. G. A. (2023). | conference paper | trust | prototype, semi-structured interviews | HCI | diabetes | healthcare practitioners | AI-CDS | Attitude |
| Bussone, A., Stumpf, S., O’Sullivan, D., Fu W.-T., Balakrishnan P., Harabagiu S., Wang F., & Srivatsava J. (2015). | conference paper | trust | user study, interviews | healthcare informatics |  | primary care practitioners | CDSS | Binary conceptualizations/ Principles |
| Chanda T, Hauser K, Hobelsberger S, Bucher TC, Garcia CN, Wies C, Kittler H, Tschandl P, Navarrete-Dechent C, Podlipnik S, Chousakos E, Crnaric I, Majstorovic J, Alhajwan L, Foreman T, Peternel S, Sarap S, Özdemir İ, Barnhill RL, … Brinker TJ. (2024). | journal article | trust | model building, reader study | health sciences, HCI | dermatology | clinicians | DNN models | Principles |
| Chen H, Ma X, Rives H, Serpedin A, Yao P, & Rameau A. (2024). | journal article | trust | experiments | health sciences | ENT (ear, nose, throat) | otolaryngologists | ML-CDST | Attitude |
| Choudhury, A., Asan, O., & Medow, J. E. (2022). | journal article | trust | survey | human factors and healthcare artificial intelligence | red blood cell transfusion without compromising organ function | healthcare professionals | CDSS/ computer-aided diagnosis (CAD) | Belief |
| Darvish, M., Holst, J.-H., Bick, M., & Bui T.X. (2024). | conference paper | trust | semi-structured interviews | HCI |  | medical & AI experts | CDSS | Binary conceptualizations |
| De Proost M & Pozzi G. (2024). | journal article | distrustworthiness, trustworthiness, trust, distrust | philosophical inquiry | philosophy |  |  |  | Relational / Structure Mechanism |
| DeCamp M & Tilburt JC. (2019). | correspondence | trust, lack of trust | position | philosophy |  |  |  | Attitude |
| Diprose WK, Buist N, Hua N, Thurier Q, Shand G, & Robinson R. (2020). | journal article | trust | survey | medical informatics |  | physicians |  | Attitude/ Principles |
| Durán JM & Jongsma KR. (2021). | journal article | trust | philosophical inquiry | philosophy |  |  |  | Binary conceptualizations |
| El-Sappagh, S., Alonso-Moral, J., Abuhmed, T., Ali, F., Bugarín-Diz, A. (2023). | journal article | trustworthiness | literature review + evaluation | computer science | Alzheimer’s disease |  | CDSS | Attitude/ Structure Mechanism |
| Ewals LJS, Heesterbeek LJJ, Yu B, van der Wulp K, Mavroeidis D, Funk M, Snijders CCP, Jacobs I, Nederend J, & Pluyter JR. (2024). | journal article | trust | experiments | health sciences | radiology | radiologists | CDSS: AI recommendations for lung nodules assessment | Binary conceptualizations |
| Fecho, M., & Zöll, A. (2023). | conference paper | trust, trustworthiness | focus group discussions, evaluation of existing applications, online survey, effectiveness test | design science |  |  | skin screening | Attitude |
| Ferlin, M., Klawikowska, Z., Niemierko, J., Grzywińska, M., Kwasigroch, A., Szurowska, E., & Grochowski, M. (2023). | book chapter | trustworthiness | prototype design | health sciences | small vessel disease |  | CDSS | Principles |
| Ferrario A, Loi M, & Viganò E. (2020). Trust does not need to be human: It is possible to trust medical AI. | response | trust, trustworthiness | philosophical inquiry | philosophy |  |  |  | Belief |
| Ferrario, A., & Loi, M. (2022). | conference paper | trust | philosophical inquiry | philosophy |  |  |  | Belief |
| Gilbank, P., Johnson-Cover, K., & Truong, T. (2020). | feature | trust | tool building, semi-structured interviews | user interface design | radiology | stakeholders from hospitals, academia, industry, and nonprofit organizations | ML radiological risk decision aid | Attitude/ Principles |
| Gille, F., Jobin, A., & Ienca, M. (2020). | journal article/position paper | trust | position/ philosophical inquiry | STS |  |  |  | Relational |
| Goel K, Sindhgatta R, Kalra S, Goel R, & Mutreja P. (2022). | journal article | trust | evaluation of different explanations, experiment | computer science | radiology | radiologists | DNN model | Belief/ Principles |
| Goh WW, Chia KY, Cheung MF, Kee KM, Lwin MO, Schulz PJ, Chen M, Wu K, Ng SS, Lui R, Ang TL, Yeoh KG, Chiu HM, Wu DC, & Sung JJ. (2024). | journal article | trust | questionnaire | health sciences | gastroenterology | gastroenterologists & gastrointestinal surgeons | computer-aided detection (CADe), computer-aided characterization (CADx), and computer-aided intervention (CADi) | Belief |
| González-Gonzalo C, Thee EF, Klaver CCW, Lee AY, Schlingemann RO, Tufail A, Verbraak F, & Sánchez CI. (2022). | journal article | trustworthiness | proposal of framework | ophthalmology |  |  |  | Principles |
| Grote, T. (2021). | commentary | trustworthiness | philosophical inquiry | philosophy |  |  |  | Relational |
| Hallowell N, Badger S, Sauerbrei A, Nellåker C, & Kerasidou A. (2022). | journal article | trust | semi-structured interviews | medical ethics |  | stakeholders (clinical geneticists, data scientists, bioinformaticians, industry and patient support group spokespersons) | computational phenotyping (CP) systems | Relational |
| Hasani N, Morris MA, Rhamim A, Summers RM, Jones E, Siegel E, & Saboury B. (2022). | journal article | trustworthiness | framework | computer science |  |  | AI-based medical imaging systems | Principles |
| Higgins O, Chalup SK, & Wilson RL. (2024). | journal article | trust | framework | HCI | nursing |  | CDSS | Structure Mechanism/ Principles |
| Högberg, C., Larsson, S., & Lång, K. (2024). | journal article | trust | survey | health sciences, information studies, STS | radiology | breast radiologists | CDSS | Relational |
| Jones C, Thornton J, & Wyatt JC. (2023). | journal article | trust, trustworthiness | Philosophical inquiry, literature review |  | clinicians |  | CDSS | Structure Mechanism |
| Katzburg O, Roimi M, Frenkel A, Ilan R, & Bitan Y. (2024). | journal article | trust | laboratory experiment, questionnaire | HCI | ICU | physicians from critical care specialties | CDSS | Attitude |
| Kerasidou A. (2021). | journal article | trust | overview | health sciences |  |  | AI healthcare tools in low-and-middle-income-countries (LMICs) | Attitude/ Relational |
| Kim, Y. J., Choi, J. H., & Fotso, G. M. N. (2024). | journal article | trust | survey | sociology, economics |  | medical professionals |  | Attitude/ Principles |
| Kim M, Sohn H, Choi S, & Kim S. (2023). | journal article | trustworthiness | literature review | healthcare informatics |  |  |  | Principles |
| Kinney M, Anastasiadou M, Naranjo-Zolotov M, & Santos V. (2024). | journal article | trust | semi-structured interviews | health sciences |  |  |  | Principles |
| Kitamura FC & Marques O. (2021). | opinion paper | trustworthiness | opinion | health science | radiology |  |  | Relational |
| Klincewicz, M., Collins, D., Jovanović, I. V., Alfano, M., & Demir-Doğuoğlu, H. (2023). | book chapter | trust | philosophical inquiry | philosophy |  |  |  | Relational/Belief |
| Kostick-Quenet K, Lang BH, Smith J, Hurley M, & Blumenthal-Barby J. (2024). | journal article | trust | semi-structured interviews | medical ethics | cardiology | patients & physicians | risk prediction for a high-stakes medical procedure: implantation of a Left Ventricular Assist Device (LVAD) for patients with advanced heart failure | Relational |
| Larasati, R., de Liddo, A., & Motta, E. (2023). | journal article | trust | interviews, prototype, survey | HCI |  | experts: machine learning technologists and medical professionals; laypeople | Explainable AI system for breast cancer assessment | Attitude |
| Lee, M. H., & Chew, C. J. (2023). | journal article | trust | experiment | HCI | occupational therapy & physiotherapy | therapists & laypersons | CDSS: rehabilitation assessment: assessing post-stroke survivors’ quality of motion | Principles |
| Lombardi, A., Marzo, S., Di Noia, T., Di Sciascio, E., Ardito, C. (2024). | conference paper | trustworthiness | interface design, questionnaire | HCI | neurology | neurology specialists | tool to diagnose Mild Cognitive Impairment (MCI) | Principles |
| Maheshwari K, Jedan C, Christiaans I, van Gijn M, Maeckelberghe E, & Plantinga M. (2024). | journal article | trust | philosophical inquiry, case study | philosophy, ethics |  | medical professionals | newborn screening | Relational |
| Naiseh, M., Al-Thani, D., Jiang, N., Ali, R., Naiseh, M., Al-Thani, D., Jiang, N., & Ali, R. (2023). | journal article | trust | within-subject study, semi-structured interviews | HCI | oncology | medical practitioners | CDSS | Principles |
| Neves BB, Omori M, Petersen A, Vered M, & Carter A. (2024). | journal article | trust | semi-structured interviews | sociology, STS | care homes | care staff, advocates, and AI developers |  | Relational |
| Nickel, P. (2022). | journal article | trust | philosophical inquiry | philosophy | clinicians |  | example of AI applications that predict re-admission to hospital | Relational |
| Pellikka PA, Hamza I, & Carter RE. (2022). | commentary | trust | position | health sciences, economics |  |  |  | Principles |
| Pickering, B. (2021). | journal article | trust | philosophical inquiry | ethics, social psychology |  |  | contact tracing | Attitude |
| Prinster, D., Mahmood, A., Saria, S., Jeudy, J., Lin, C. T., Yi, P. H., & Huang, C.-M. (2024). | journal article | trust | multicenter, prospective randomized study | computer science | radiology | physicians |  | Binary conceptualizations |
| Procter, R., Tolmie, P., Rouncefield, M. (2023). | journal article | trustworthiness | ethnographic case studies | HCI |  | multi-disciplinary team | breast cancer screening | Principles/ Structure Mechanism |
| Rainey, C., Bond, R., McConnell, J., Hughes, C., Kumar, D., & McFadden, S. (2024). | journal article | trust | experiment: interpreting radiographic examinations | implementation science | radiology | radiographers |  | Principles |
| Rey, A., & Bouaynaya, W. (2022). | conference paper | trust | ethnographic study: participant observation & semi-structured interviews | management? |  |  |  | Structure Mechanism |
| Rojas JC, Teran M, & Umscheid CA. (2023). | journal article | trustworthiness, trust | lit review? |  | ICU | intensivists | CDSS | Principles |
| Saboury B, Bradshaw T, Boellaard R, Buvat I, Dutta J, Hatt M, Jha AK, Li Q, Liu C, McMeekin H, Morris MA, Scott PJH, Siegel E, Sunderland JJ, Pandit-Taskar N, Wahl RL, Zuehlsdorff S, & Rahmim A. (2023). | journal article | trustworthiness | strategic plan and a summary of the deliberations of the SNMMI AI task force |  |  |  |  | Structure Mechanism |
| Schwartz JM, George M, Rossetti SC, Dykes PC, Minshall SR, Lucas E, & Cato KD. (2022). | journal article | trust | semi-structured interviews | HCI | acute and intensive care units, excluding pediatric, neonatal, hospice, emergency, oncology, labor and delivery, behavioral or psychiatric, observational, perioperative, same-day surgery, and plastic surgery units | clinicians: physicians & nurses | CDSS | Belief |
| Segers, S., & Mertes, H. (2022). | journal article | trust, trustworthiness | philosophical inquiry | ethics, philosophy |  | patients |  | Structure Mechanism |
| Shamszare, H., & Choudhury, A. (2023). | journal article | trust | survey | health sciences | most common clinical expertise areas were family medicine (17%), geriatrics (17%), and pediatrics (11%) | healthcare professionals |  | Attitude |
| Shevtsova D, Ahmed A, Boot IWA, Sanges C, Hudecek M, Jacobs JJL, Hort S, & Vrijhoef HJM. (2024). | journal article | trust | rapid review, electronic survey | implementation science |  |  |  | Belief |
| Silcox, C., Dentzer, S., & Bates, D. W. (2020). | journal article | trust | checklist | health sciences |  | clinicians | CDSS | Principles |
| Sivaraman, V., Bukowski, L., Levin, J., Kahn, J., Perer, A., Sivaraman, V., Bukowski, L. A., Levin, J., Kahn, J. M., & Perer, A. G. A. (2023). | conference paper | trust | development of CDSS & think-aloud study, semi-structured interview | health informatics | ICU | intensive care clinicians | CDSS | Attitude |
| Smith, M. W., Kalsy, M., Weir, C. R., Brown, C. R., Virani, S. S., & Garvin, J. H. (2023). | conference paper | trust | prototype development & think-aloud sessions | health sciences | cardiology | 4 PCPs (one Nurse Practitioner, three physicians); four clinical pharmacists, one Registered Nurse care manager | CDSS | Structure Mechanism |
| Sperling J, Welsh W, Haseley E, Quenstedt S, Muhigaba PB, Brown A, Ephraim P, Shafi T, Waitzkin M, Casarett D, & Goldstein BA. (2025). | journal article | trust | focus groups & individual interviews | medical informatics |  |  |  | Principles |
| Starke G & Ienca M. (2024). | journal article | mistrust, distrust | philosophical inquiry | medical ethics |  |  |  | Principles/ Binary conceptualizations |
| Starke G, van den Brule R, Elger BS, & Haselager P. (2022). | journal article | trust | philosophical inquiry | human– robot interactions, psychology and sociology |  |  |  | Structure Mechanism/ Attitude |
| Strange, M., Ericson P., Khairova N., & De Vos M. (2024). | workshop paper | trust | philosophical/theoretical inquiry | political theory & Coactive Design |  |  |  | Relational |
| Szabo L, Raisi-Estabragh Z, Salih A, McCracken C, Ruiz Pujadas E, Gkontra P, Kiss M, Maurovich-Horvath P, Vago H, Merkely B, Lee AM, Lekadir K, & Petersen SE. (2022). | journal article | trustworthiness, trust | narrative review, framework | data science, health science | cardiology |  | (1) image acquisition and reconstruction— which helps to reduce the scan time, (2) improving the imaging workflow and efficiency of time-expensive tasks such as segmentation, (3) improving the diagnosis-making process, (4) evaluation of disease progression and prognosis, (5) assessment of treatment effectiveness, and (6) generation of new knowledge | Principles |
| van der Zander QEW, Roumans R, Kusters CHJ, Dehghani N, Masclee AAM, de With PHN, van der Sommen F, Snijders CCP, & Schoon EJ. (2024). | journal article | trust | experiment, optional interviews | health sciences | clinical endoscopy | endoscopists | CADx | Attitude |
| Wallace PJ. (2024). | commentary | trust | commentary/ introduction? | health sciences |  |  |  | Principles |
| Winter, P., & Carusi, A. (2022). | journal article | trust | case study: qualitative interviews | STS | pulmonary hypertension (PH) | two PH clinicians, one consultant PH nurse, one radiologist, one computer scientist, one data scientist, and one biomedical scientist | AI algorithms for the early diagnosis of PH | Relational |
| Wolkenstein A. (2024). | journal article | mistrust, distrust | philosophical inquiry | ethics |  |  | medical black-box algorithms |  |
| Wünn, T., Sent, D., Peute, L. W. P., Leijnen, S., Nowaczyk S., Biecek P., Chung N.C., Vallati M., Skruch P., Jaworek-Korjakowska J., Parkinson S., Nikitas A., Atzmüller M., Kliegr T., Schmid U., Bobek S., Lavrac N., Peeters M., van Dierendonck R., … Dimitrova V. (2024). | workshop paper | trust | prototype design, questionnaire | medical informatics |  |  |  | Attitude |
| Yang, Q., Hao, Y., Quan, K., Yang, S., Zhao, Y., Kuleshov, V., Wang, F., Yang, Q., Hao, Y., Quan, K., Yang, S., Zhao, Y., Kuleshov, V., & Wang, F. G. A. (2023). | conference paper | trust | semi-structured interviews, prototype design | HCI, biomedical sciences |  | clinicians & their assistants | Clinical decision support tools (DSTs) | Belief |
| Zhang J & Zhang ZM. (2023). | journal article | trustworthiness, trust | framework, philosophical inquiry | medical informatics |  |  |  | Belief |
| Zuchowski LC, Zuchowski ML, & Nagel E. (2024). | journal article | trust | framework | health sciences |  | medical professionals, patients |  | Belief |

Table of Trust Disruptions

| **Trust disruption** | **Number** | **References** |
| --- | --- | --- |
| (Healthy) skepticism | 9 | Asan et al. (2020)  Chen et al. (2024)  Choudhury et al. (2022)  De Proost & Pozzi (2024)  Fecho & Zöll (2023)  Kerasidou (2021)  Lombardi et al. (2024)  Strange et al. (2024)  Winter & Carusi (2022) |
| Betrayal of trust | 1 | Zuchowski et al. (2024) |
| Blind trust | 5 | Branley-Bell et al. (2020)  Bussone et al. (2015)  Choudhury et al. (2022)  Högberg et al. (2024)  Segers & Mertes (2022) |
| Distrust | 17 | Brown et al. (2024)  Bussone et al. (2015)  De Proost & Pozzi (2024)  Hallowell et al. (2022)  Katzburg et al. (2024)  Kerasidou (2021)  Kinney et al. (2024)  Kostick-Quenet et al. (2024)  Larasati (2023)  Maheshwari et al. (2024)  Neves et al. (2024)  Pickering (2021)  Rojas et al. (2023)  Starke & Ienca (2024)  Strange et al. (2024)  Winter & Carusi (2022)  Wolkenstein (2024) |
| Distrustworthiness | 1 | De Proost & Pozzi (2024) |
| Misplaced distrust | 1 | Starke & Ienca (2024) |
| Misplaced trust | 2 | Bürger et al. (2024)  Starke & Ienca (2024) |
| Mistrust | 2 | Saboury et al. (2023)  Wolkenstein (2024) |
| No trust/lack of trust | 5 | DeCamp & Tilburt (2019)  Diprose et al. (2020)  Hallowell et al. (2022)  Högberg et al. (2024)  Winter & Carusi (2022) |
| Over- & Undertrust | 11 | Browne et al. (2022)  Choudhury et al. (2022)  Darvish et al. (2024)  Högberg et al. (2024)  Kostick-Quenet et al. (2024)  Larasati et al. (2023)  Naiseh et al. (2023)  Nickel (2022)  Pellikka et al. (2022)  Rojas et al. (2023)  van der Zander et al. (2024) |
| Trust calibration mistake | 1 | Naiseh et al. (2023) |
| Trust repair | 1 | Pickering (2021) |
| Untrustworthiness | 4 | Baldassarre et al. (2024)  Jones et al. (2023)  Kim et al. (2023)  Starke & Ienca (2024) |
